# Prospective long-term evaluation of incomplete distal renal tubular acidosis in idiopathic calcium nephrolithiasis diagnosed by low-dose NH_4_CL loading – gender prevalences and impact of alkali treatment

**DOI:** 10.1007/s40620-021-01207-7

**Published:** 2022-01-01

**Authors:** Juri Sromicki, Georg Kacl, Malin Föhl, Bernhard Hess

**Affiliations:** 1 Internal Medicine and Nephrology, Kidney Stone Center Zurich, Klinik Im Park and University of Zurich, Bellariastrasse 38, CH-8038 Zurich, Switzerland; 2 Department of Cardiac Surgery, University Hospital, Zurich, Switzerland; 3 Radiology, Klinik Im Park, Zurich, Switzerland

**Keywords:** Incomplete distal renal tubular acidosis in idiopathic calcium nephrolithiasis, Gender prevalences, Intrarenal calcifications, Nephrocalcinosis, Effects of alkali treatment

## Abstract

**Purpose:**

Prospective evaluation of the prevalence of incomplete distal renal tubular acidosis (idRTA) in idiopathic calcium stone formers (ICSF) diagnosed by half-dose ammonium chloride loading (NH_4_Cl, 0.05 g/kg body weight/day) and impact of alkali treatment of idRTA.

**Methods:**

Evaluation of 386 consecutive idiopathic calcium stone formers (ICSF) (280 males, 106 females) for idRTA. If screening fasting urine pH was > 5.80, 1-day NH_4_Cl loading was performed without severe adverse effects. Normally, urine pH falls below 5.45.

**Results:**

Sixty-four idiopathic calcium stone formers exhibited idRTA, one complete dRTA. Prevalence was higher in women (25.4%) than in men (13.6%). Thus, for more equilibrated comparisons, we formed pairs of 62 idiopathic calcium stone formers (ICSF) with and 62 without idRTA, matched for gender, age, BMI and serum creatinine. Idiopathic calcium stone formers with idRTA more often had hypercalciuria (*p* < 0.025) and urine citrate < 2 mmol/d (*p* < 0.05), formed calcium phosphate stones more frequently, exhibited higher numbers of stones/year (1.4 ± 1.5 vs. 0.9 ± 0.8, *p* = 0.034) and 2.5 times more intrarenal calcifications (4.6 ± 5.9 vs. 1.8 ± 3.6, *p* = 0.002). All idiopathic calcium stone formers with idRTA were recommended chronic alkali therapy. After 4–15 years of follow-up, stone events /years follow-up (stone passage or urologic intervention) were higher in patients non-adherent to alkali therapy (0.61 ± 0.92) than in patients adherent to treatment (0.11 ± 0.21, *p* = 0.006).

**Conclusion:**

Incomplete distal renal tubular acidosis is 1.8-fold more prevalent among female idiopathic calcium stone formers, predicts more stone recurrences, predisposes to calcium phosphate stones and is associated with 2.5 times more intrarenal calcifications vs. non-idRTA patients. Chronic alkali treatment reduces clinical stone recurrences by 5.5 times.

**Graphical abstract:**

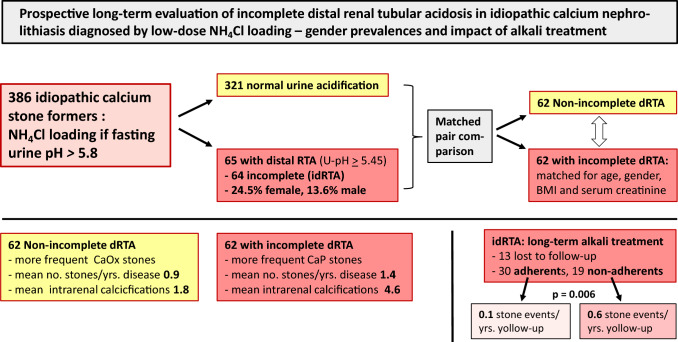

## Introduction

On a diet rich in animal proteins, the kidneys have to excrete up to 100 mmol of hydrogen ions per day [1]. Reduced H^+^ secretion by collecting tubules causes distal renal tubular acidosis (dRTA) [2–4], either *complete* (overt metabolic acidosis, elevated urine pH) or more frequently *incomplete* (idRTA, no systemic acidosis, elevated urine pH) [2, 4, 5]. In both forms, chronic H^+^ retention predisposes to calcium nephrolithiasis [2–4] and low bone mass [6–8].

The routine diagnosis of idRTA is based on urine pH measurements after a *defined acid load*, originally described by Wrong and Davies [9]. They arbitrarily loaded patients with 0.1 g ammonium chloride (NH_4_Cl) per kg of body weight which was considered to produce maximally acid urine. An inability to lower urine pH below 5.3 without systemic acidosis signifies a “subtle” defect in distal tubular H^+^ secretion [9]. However, the high NH_4_Cl dose applied often causes gastric irritation and vomiting [5, 10]. Alternatively, administration of furosemide and fludrocortisone with subsequent urine pH measurements produces equal results with better tolerance [5, 10].

We previously introduced lower NH_4_Cl loading (0.05 g/kg body weight) over 3 days [11, 12]. This never caused vomiting, and the drop in urine pH matched the results of the classic test, although a formal direct comparison of both protocols was not performed [11]. For practical reasons, we later developed a 1-day test with the same NH_4_Cl load in 3 doses before main meals. In healthy controls, urine pH fell by 0.62 units to below 5.45 without systemic acidosis [8]. This NH_4_Cl test was applied in the present prospective 11-year study in order to establish gender prevalences of idRTA among unselected consecutive idiopathic calcium stone formers (ICSF). Moreover, we studied gender-matched pairs of idiopathic calcium stone formers (ICSF) with and without idRTA and assessed the impact of alkali treatment on stone recurrences in those with idRTA.

## Subjects and methods

### Whole stone-former cohort 2006–2016

From 2006 until 2016, 531 consecutive renal stone formers (380 men, 151 women), aged 17 to 82 years, were referred to our Kidney Stone Center by hospital or practicing urologists for metabolic evaluation.

### Idiopathic calcium stone formers (ICSF)

There were 451 calcium stone formers, 65 of whom were excluded due to secondary causes (bariatric surgery, primary hyperparathyroidism, Crohn’s disease/colitis ulcerosa, medullary sponge kidney, treatment with carboanhydrase inhibitors or HIV medication). Thus, 386 patients remained, none of whom had a history of autoimmune disorders or had received immunomodulatory medication, and therefore were classified as idiopathic calcium stone formers (ICSF).

### Matched-pair substudy

For more equilibrated comparisons due to the strong female preponderance among idRTA patients (results), we formed 62 pairs of idiopathic calcium stone formers (ICSF) with and without idRTA, matched for gender, age, BMI and serum creatinine. Eleven idiopathic calcium stone formers (ICSF) with idRTA and positive family history for nephrolithiasis were also part of a recent genetic study [13].

## Laboratory evaluation

### Stone clinic procedure

According to our protocol [14], patients had dinner by 7:00 p.m., as alkali-rich late dinners could falsely raise urine pH [15]. Patients did not drink after midnight. The next day, first urine was voided at home, before fasting blood and urine were obtained at the stone center [8, 14]. Fasting whole blood was analyzed for ionized calcium, sodium, potassium, urea and creatinine (blood gas analyzer, i-STAT) and for phosphate, 25-OH-vitamin D and parathyroid hormone (UNILABS, Dübendorf/Switzerland). Urine was tested for infection (dipstick), and pH was measured by ion-selective electrode (Metrohm 744 pH meter).

### 24 h-urine

On free-choice diet, patients twice collected 24 h-urines. Routine chemistries were determined by autoanalyzer, and oxalate and citrate by ion chromatography. Creatinine clearance was calculated [16]. Prevalence of 24 h-urine abnormalities was assessed using normal values obtained from healthy Swiss volunteers [14] (see Table 1). Normal urine volume was defined as ≥ 2,000 ml/d [17]. Intake of salt and total protein was calculated from 24-h urine sodium and urea [18] as follows:

**Table 1:** Prevalence of major pathophysiologic risk factors among 386 consecutive idiopathic calcium stone formers (ICSF) 2006–2016

Parameter	Prevalence overall	Prevalence 280 men	Prevalence 106 women	*P* value (c2) men vs. women
COM stones	190 (49.2%)	152 (54.3%)	38 (35.9%)	< 0.0001
COD stones	37 (9.6%)	24 (8.6%)	13 (12.3%)	< 0.025
CaP stones	15 (3.9%)	9 (3.2%)	6 (5.7%)	NS
COM/COD stones 50%/50%	10 (2.6%)	9 (3.2%)	1 (0.9%)	< 0.05
CaOx/CaP stones	48 (12.4%)	33 (11.8%)	15 (14.2%)	NS
Calcium (radiopaque) stones	86 (22.3%)	53 (18.9%)	33 (31.1%)	< 0001
Protein intake (> 1 g/kg/d)	265 (68.7%)	201 (71.8%)	64 (60.4%)	< 0.001
Low volume (≤ 2000 ml/d)	197 (51.0%)	146 (52.1%)	51 (48.1%)	NS
Pattern of idRTA/dRTA	65 (16.8%)	38 (13.6%)	27 (25.5%) (1 complete dRTA)	< 0.001
Hypocitraturia (< 1.7 (m)/ < 1.9 (f) mmol/d)	63 (16.3%)	41 (14.6%)	22 (20.8%)	< 0.025
Hypercalciuria (> 9 (m)/ > 8 (f) mmol/d)	60 (15.5%)	41 (14.6%)	19 (17.9%)	NS
Hyperoxaluria (> 0.44 mmol/d)	42 (10.9%)	37 (13.2%)	5 (4.7%)	< 0.0001
Hyperuricosuria (> 5 (m)/ > 4 (f) mmol/d)	32 (8.3%)	25 (8. 9%)	7 (6.6%)	NS

Normal values are based on 24 h-urine excretion rates previously obtained from 103 male and 73 healthy female Swiss volunteers on a habitual Swiss diet [14]. *ICSF* idiopathic calcium stone formers

- Urine-Na (mmol/d) × 0.058 = grams salt/day.

- [Urine-Urea × 0.18] + 13 = grams protein/day. This value was normalized to kilograms normal body weight by the Broca index (Height (cm) - 100 = kg normal weight).

Urinary calcium oxalate supersaturation in 124 idiopathic calcium stone formers (ICSF) (matched-pair substudy) was calculated using Tiselius’ AP (CaOx) index EQ [19].

### Fasting morning urine pH measurements and NH_4_Cl loading

Fasting urine pH (normal ≤ 5.80) was measured at the stone clinic (pH meter) and by idiopathic calcium stone formers (ICSF) at home (fine-graded test strips). If urine pH was ≥ 5.8 in 4 out of 5 measurements, patients underwent *1-day NH*_*4*_*Cl loading* [8]. This procedure had been approved by the Ethics Committee of the Canton of Zurich [8]. Patients ingested NH_4_Cl, 0.05 g/kg body weight/day, in 400 mg-capsules, 3 doses/day at home, 25 min before meals. After an overnight fast of 12 h, second morning fasting urine and venous blood were analyzed. Normal values are urine pH < 5.45 and venous bicarbonate > 20.5 mmol/l [8]. NH_4_Cl was generally well tolerated; 1/3 of patients reported slight nausea, but never vomiting.

## Stones per patient and stone types

*Stones per patient* were defined as the sum of reported spontaneous stone passages *plus* the number of urologic interventions (URS, ESWL, open surgery) *plus* the number of loose stones remaining in kidney pelvis/calyces on CT scans or X-rays.

Results of *stone analyses* (X-ray diffraction or infrared spectroscopy) were either provided by referring urologists or obtained from stone specimens directly delivered to us. Stone types were named according to the major component, if this amounted to ≥ 65%, or were categorized as mixed stones. If stone analysis was unavailable, stones were categorized as “calcium” or “non-calcium” according to X-ray or CT scans.

### Imaging studies

For initial evaluation, CT scans obtained from referring urologists were mainly used. If CT scans were missing, they were performed at our institution. For the matched-pair subanalysis, an experienced uroradiologist (G.K.), unaware of the patients’ metabolism, re-screened all available CT scans at the time of referral and counted the number of tissue calcifications as well as loose kidney stones in both kidneys/ureters.

### Treatments and follow-up

All idiopathic calcium stone formers (ICSF) received 20 min of dietary recommendations (B.H.), individualized according to personal preferences (evident from diet histories). The principles consisted of significantly increasing fluid intake, raising calcium consumption *with* meals/snacks in order to reduce intestinal oxalate uptake, and equilibrating dietary acid–base balance by reducing meat protein to one serving per day while ingesting at least 3 servings of alkali (vegetables, salad, fruit) [20]. This strategy significantly reduces urinary calcium oxalate supersaturation [20]. After 3–4 months, control 24 h-urine and dietary protocols were obtained and discussed with the patients before they were sent back to their referring physicians/hospitals. Further controls at our stone center were only carried out upon specific request.

Among the 62 matched pairs, those with idRTA, but not those without, were strongly recommended and prescribed long-term potassium citrate (40–60 meq/day) besides dietary measures. Between 4 and 15 years after treatment initiation, idRTA patients were contacted by phone (B.H.) in early 2021 and asked (1) whether they had been adherent to alkali until the phone call; and (2) how many spontaneous stone passages or urologic interventions had occurred since the therapeutic recommendation. Accordingly, idRTA patients were retrospectively subdivided into adherents vs. non-adherents to alkali.

### Statistics

All data are means ± SD. For 24 h urine, mean values of 2 collections were used. Comparisons within (paired *t* test) and between (unpaired *t* test) groups were performed by Student’s *t* test. Frequencies of features between 2 groups were compared using Chi square statistics. Selection of matched pairs of idiopathic calcium stone formers (ICSF) was performed using IBM SPSS statistics for Windows, Version 25 (IBM Corp., Armonk NY, USA). *p* values < 0.05 were considered statistically significant.

## Results

### Whole stone former cohort

Body mass index (BMI) was 27.0 ± 4.6 kg/m^2^, and 22% of stone formers were obese (BMI ≥ 30 kg/m^2^, 21.6% men, 23.0% women). Recurrent stone formation was present in 89.5%, and 247 patients (46.5%) had a positive family history. Mean number of stones was 7.8 ± 13.8 (range 1–156). Stone number was much higher in cystinurics (26.6 ± 32.8 stones). Stone types were 84.9% calcium, 10.4% uric acid, 1.5% mixed calcium oxalate/uric acid, 1.5% infection and 1.7% cystine stones.

### Idiopathic calcium stone formers ICSF (*n* = 386)

**Fig. 1:**
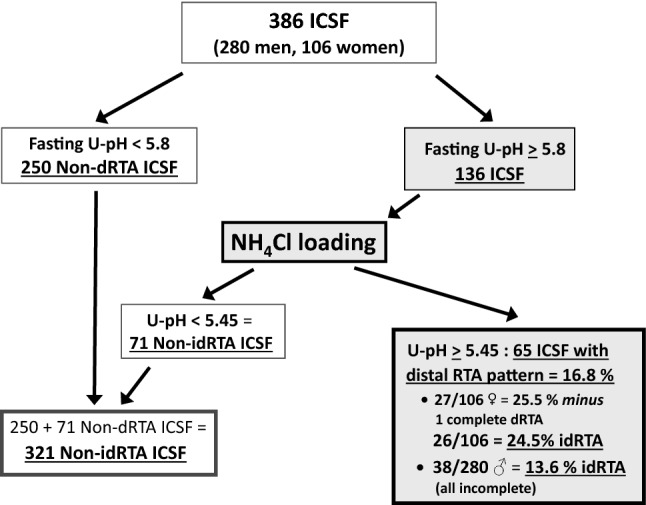
Flow diagram of fasting urine pH screening and ammonium chloride loading in 386 consecutive idiopathic calcium kidney stone formers (ICSF) 2006–2016. NH_4_Cl = ammonium chloride, *dRTA* distal renal tubular acidosis, *idRTA* incomplete distal renal tubular acidosis. Prevalence of idRTA in women amounts to 24.5% (26 out of 106 after deduction of 1 patient with *complete* dRTA)

Figure 1 shows that fasting urine pH remained ≥ 5.80 in 136 out of 386 ICSF who thus underwent NH_4_Cl loading: acidification was normal in 71 cases, but 65 failed to lower pH, indicating disturbed distal renal-tubular acidification. With venous bicarbonate remaining within normal limits, 64 patients had incomplete dRTA, and only one female idiopathic calcium stone formers (ICSF) had *complete* dRTA. Overall, idRTA was the third-most common stone risk factor among idiopathic calcium stone formers (ICSF).

The prevalence of *incomplete* dRTA in women is almost double that in men (Fig. 1). In comparison with Non-idRTA patients, idiopathic calcium stone formers (ICSF) with idRTA were younger (44 ± 16 vs. 51 ± 18 years, *p* = 0.0002) and more often had a positive family history (52 vs. 47%, *p* < 0.05). Blood parameters showed lower serum potassium in idRTA patients (3.91 ± 0.32 vs. 4.00 ± 0.36, *p* = 0.016). When comparing the 71 idiopathic calcium stone formers (ICSF) without idRTA who underwent NH_4_Cl loading (see Fig. 1*)* to those with manifest idRTA, venous bicarbonate before (27.68 ± 2.56 mmol/l with *vs.* 27.42 ± 4.50 without idRTA) as well as after acid loading (26.55 ± 2.56 mmol/l with *vs.* 26.12 ± 3.78 mmol/l without idRTA) did not differ between groups. A significant decrease in venous bicarbonate after acid loading was noted, both in patients with idRTA (− 1.13 ± 2.32 mmol/l, *p* = 0.0002 vs. before) and in those without (− 1.30 ± 3.96 mmol/l, *p* < 0.00001 vs. before), without group differences. Venous pH before (7.364 ± 0.040 with *vs.* 7.358 ± 0.054 without idRTA) and after acid loading (7.357 ± 0.048 with *vs.* 7.347 ± 0.054 without dRTA) was also not different between groups.

While 24-h urinary oxalate, uric acid, urea, phosphate, citrate and magnesium did not differ between idRTA and Non-idRTA patients (data not shown), idiopathic calcium stone formers (ICSF) with idRTA exhibited higher 24-h urinary calcium excretion (7.0 ± 3.2 vs. 5.9 ± 1.8 mmol/d, *p* = 0.003). This was not explained by differences in sodium and protein consumption, known modifiers of calciuria [21].

## Matched-pair substudy

### Clinical and laboratory data

Of the 64 idiopathic calcium stone formers (ICSF) with idRTA, 62 could be matched to 62 Non-idRTA patients for gender (26 women/36 men in each group), age (44 ± 16 *vs*. 44 ± 16 years), BMI (24.7 ± 4.0 *vs.* 24.2 ± 3.2 kg/m^2^), and serum creatinine (77 ± 16 *vs*. 82 ± 24 μmol/l). Venous bicarbonate, Ca^++^, 25-OH-vitamin D and creatinine clearance were not different between groups (data not shown). By definition, fasting urine pH was higher in idRTA patients, both before (6.45 ± 0.47 *vs.* 6.08 ± 1.03, *p* = 0.05) and after NH_4_Cl (5.93 ± 0.47 *vs.* 5.17 ± 0.40, *p* = 0.0002). As depicted in Table 2, hypercalciuria and low citrate (< 2.0 mmol/24 h) were more common among idRTA patients. All other 24 h-urine parameters were not different between groups, nor was calcium oxalate supersaturation (0.91 ± 0.47 with idRTA *vs*. 0.89 ± 0.55 without, *p* = 0.65).

**Table 2:** Main features of idiopathic calcium stone formers (ICSF) with idRTA and without idRTA, matched for age, gender, body-mass index and serum creatinine

Parameters	idRTA idiopathic calcium stone formers (ICSF) (62)	Matched idiopathic calcium stone formers (ICSF) (62)	*p* value
Hypercalciuria	15/62 (24.2%)	8/62 (12.9%)	< 0.025
24 h-urine citrate < 2.0 mmol/d	20 /62 (32.3%)	14/62 (22.6%)	< 0.05
Pure CaOx stones	22/62 (35.5%)	38/62 (61.3%)	< 0.0005
Stones with CaP	23/62 (37.0%)	5/62 (8.1%)	< 0.0001
Stones/years of disease	1.36 ± 1.50	0.92 ± 0.79	0.034
Loose stones/idiopathic calcium stone formers (ICSF) (radiologic findings)	0.79 ± 1.50	0.82 ± 1.11	NS
idiopathic calcium stone formers (ICSF) with intrarenal calcifications	42/57 (73.7%)	29/55 (52.7%)	< 0.005
Intrarenal calcifi- cations/patient	4.61 ± 5.85	1.84 ± 3.63	0.002

*idRTA* incomplete distal renal tubular acidosis, *ICSF* idiopathic calcium stone formers. For details, see text

### Associated pathologies, stone history, stone types and radiological stone burden

**Fig. 2:**
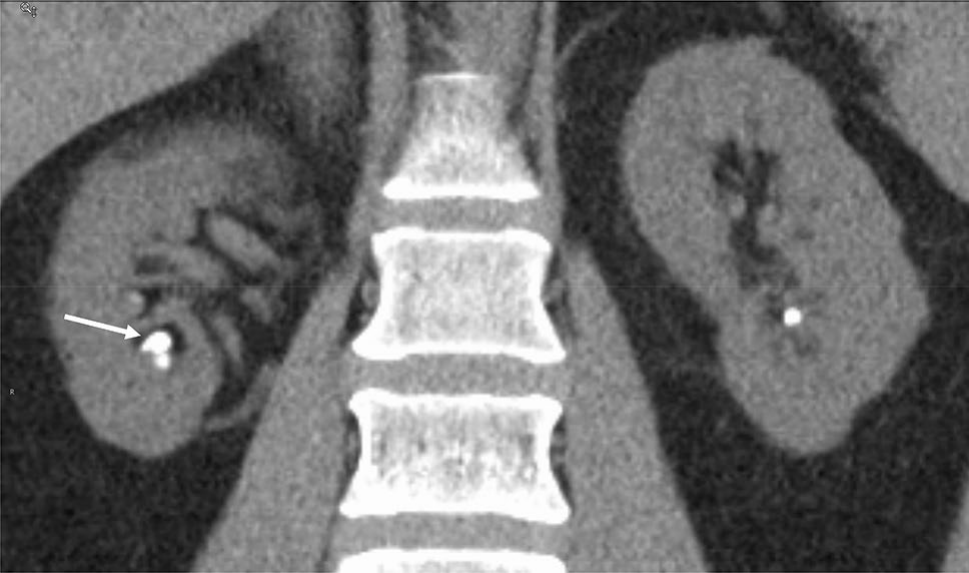
CT scan demonstrating a loose intracalyceal stone in the right kidney (arrow) and papillary calcifications in both kidneys

Patients with idRTA rather had formed apatite/brushite, but more rarely calcium oxalate stones (Table 2). Numbers of stones per years of disease were higher in idRTA patients, indicating *more active disease.* Finally, initial CT scans were available for 57 out of 62 idiopathic calcium stone formers (ICSF) with and 55 out of 62 without idRTA for re-evaluation. Figure 2 distinguishes a small calyceal loose stone from bilateral intrarenal (papillary) calcifications. At equal numbers of loose stones, idRTA patients exhibited 2.5-fold more intrarenal calcifications (Table 2).

### Impact of long-term alkali treatment

We lost 13 idRTA patients to follow-up. Of the remaining 49 idRTA patients, 30 were still adherent to alkali 4–15 years after treatment initiation, but 19 had stopped or never taken medication. After the phone contact, two female non-adherents returned to our stone clinic: transition from idRTA (venous bicarbonate 23.8 mmol/l in 2009) into mild complete dRTA (venous bicarbonate 20.3 mmol/1 in 2021) in one, and a tremendous increase in the number of intrarenal calcifications on CT (from 15 in 2009 to 48) in the other non-adherent female were noted.

At equal follow-up time periods (6.9 ± 3.2 years in adherents vs. 7.2 ± 2.9 years in non-adherents), 53% (10/19) of non-adherents to alkali reported clinical stone events during follow-up vs. 33% (10/30) of adherents (*p* < 0.05). Moreover, clinical stone event numbers per years of follow-up were 5.5-fold higher in non-adherents (0.61 ± 0.92, range 0–3.33/year) than in adherents to alkali (0.11 ± 0.21, range 0–1.33/year, *p* = 0.006).

## Discussion

The present study represents the largest prospective long-term evaluation for idRTA in non-selected idiopathic calcium stone formers (ICSF) to date. Four key findings emerge: first, NH_4_Cl loading at half the “classic” dose can be performed at home with results equal to those originally published [9]; second, incomplete idRTA is highly prevalent, especially in women; third, idRTA patients exhibit more active stone disease and more marked nephrocalcinosis; and fourth, alkali therapy reduces clinical stone recurrences in idRTA patients.

The original NH_4_Cl loading test requires serial blood and urine measurements in a hospital unit (5, 9, 10), and the high NH_4_Cl dose often causes unpleasant gastrointestinal side effects [5, 10]. The key criteria for diagnosing idRTA is the drop in urine pH [9]. In the 10 normals of the original test, average urine pH had fallen by 0.7 units, from about 5.5 (extrapolated from Fig. 1 in [9]) to 4.81. After lower-dose NH_4_Cl loading, our 21 healthy controls lowered their average urine pH by 0.62 units (5.71–5.09) [8], and Non-idRTA patients in the present matched-pair substudy reduced mean pH by even 0.91 units (6.08–5.17). Moreover, our controls exhibited an acidemic reaction (blood pH fell by 0.03 units) and a drop in urinary citrate, a sensitive marker of renal intracellular acidosis [8]. Thus, our better tolerated procedure appears to be a valid alternative for screening subtle defects in distal tubular acidification. In addition, the test can be performed at home and thus is more practical for outpatient evaluation which may be important at times of restricted health care budgets.

Widely variable prevalences between 2.3% and 31% [22, 23] have been described for idRTA. Our overall prevalence of 16.8% matches the 13.7% that others have found in stone formers of both genders [24]. The prevalence was unexpectedly high in women, as every fourth female idiopathic calcium stone formers (ICSF) exhibits idRTA. This matches the findings in patients with low bone mass [8] and resembles earlier findings [2, 22, 23]. The reason for this remains unclear, also because autoimmune disorders—more common in females and predisposing to idRTA—were not represented among our idiopathic calcium stone formers (ICSF). However, our findings certainly explain why studies exclusively [12] or mainly [5] investigating men found lower idRTA prevalences.

We confirm that idRTA patients are younger [22, 24, 25], and more often have a positive family history of stones [22] and lower serum potassium [5, 24]. The higher urinary calcium in stone formers with idRTA, not explained by higher salt and protein intake, most likely is due to idRTA itself, as chronic acid retention raises calciuria by combined effects of calcium mobilization from bone and reduced renal tubular calcium reabsorption [21, 26].

We find that idiopathic calcium stone formers (ICSF) with idRTA exhibit more active disease. Intuitively, the increase in stone activity by almost 50% must relate to the retention of H^+^ ions which permanently favors urinary calcium phosphate crystallization by elevated urine pH, increased calciuria and reduced urinary citrate excretion, as intracellular acidosis induces a rise in citrate uptake by tubular cells [4]. Altogether, idRTA represents a subclinical acidotic or “preacidotic” [5] state which manifests initially with eubicarbonatemic H + -retention [27] and may progress to mild complete dRTA, as indicated by one of our idiopathic calcium stone formers (ICSF) (see results). Indeed, a recent analysis has nicely demonstrated that idRTA apparently is a continuous trait [5] which may allow for transitions to overt dRTA over time. The abnormality might be either acquired or genetic [28]. Evidence for the latter, however, was only found in 10% of idRTA patients with positive family history for stones in a recent genetic study [13].

We demonstrate that idRTA stone formers exhibit more pronounced nephrocalcinosis. Previously, nephrocalcinosis was found in 29% of distal RTA patients [29]. The CT scan analysis in our highly active idiopathic calcium stone formers (ICSF) revealed 74% calcifications in idRTA patients, and the number of calcifications were 2.5-fold higher than in Non-idRTA patients. It appears conceivable that nephrocalcinosis is a consequence of idRTA, since chronic H^+^ retention increases the release of calcium and phosphate from bone into blood which may promote intrarenal calcifications [26], as impressively demonstrated by one of our idRTA patients never taking alkali (see results). On the other hand, preexisting calcifications, for instance following pyelonephritic tissue necrosis, might secondarily impair renal H^+^ excretion.

An important finding is the effect of long-term alkali treatment in idRTA patients. Chronic alkali treatment, well-known to reduce stone recurrences also in Non-idRTA-ICSF, is considered the cornerstone of therapy in idRTA [28], although prospective studies on alkali treatment are rare. In 9 highly active stone formers with idRTA, potassium citrate completely inhibited new stone formation [30]. Our retrospective comparison of alkali-adhering to non-adhering idRTA patients demonstrates that clinical stone recurrences are 5.5-times less frequent in those adhering to long-term alkali treatment. The most important long-term effect of alkali therapy, however, might be the inhibition of progressive nephrocalcinosis.

Limitations of the study are that, although data had been collected prospectively, we retrospectively re-analyzed endpoints such as number of stones and calcifications on CT scans. In addition, patients with idRTA were not prospectively randomized to alkali treatment vs. no alkali, but only subdivided into adherents vs. non-adherents to alkali with respective clinical outcomes, based on personal phone interviews.

In conclusion, this study establishes key features of idRTA (summarized above). In idiopathic calcium stone formers (ICSF with hypercalciuria, low urinary citrate and fasting morning urine pH > 5.8 as well as apatite or brushite stones, searching for idRTA seems mandatory. The high prevalence of idRTA among I idiopathic calcium stone formers (CSF), the higher activity of stone disease and the more marked nephrocalcinosis in those with idRTA as well as the beneficial effect of alkali treatment on clinical stone recurrences raise the question whether or not calcium stone formers with idRTA should still be classified as “idiopathic”.
